# Standardized sample preparation of paediatric bronchoalveolar lavage fluid for mass spectrometry based proteomic analysis

**DOI:** 10.1186/s40348-025-00205-0

**Published:** 2025-11-20

**Authors:** Nadine Freitag, Dirk Schramm, Anja Stefanski, Christina B. Schroeter, Kai Stühler, Gereon Poschmann, Marc D. Driessen

**Affiliations:** 1 Department of General Pediatrics, Neonatology and Pediatric Cardiology, Medical Faculty, University Hospital Düsseldorf, Heinrich Heine University, Düsseldorf, Germany; 2 Molecular Proteomics Laboratory, BMFZ, Heinrich Heine University Düsseldorf, Düsseldorf, 40225 Germany; 3 Department of Cell Biology, Harvard Medical School, Boston, MA 02115 USA; 4 Department of Neurology, Medical Faculty, University Hospital Düsseldorf, Heinrich Heine University, Düsseldorf, Germany; 5 Institute of Molecular Medicine, Proteome research, Medical Faculty, University Hospital, Heinrich Heine University Düsseldorf, Düsseldorf, 40225 Germany; 6 Faculty of Medicine and University Hospital Cologne, Center for Dental, Oral and Maxillofacial Medicine (central facilities), University of Cologne, Cologne, Germany

**Keywords:** Pulmonary proteome, Bronchoalveolar lavage fluid, LC-MS/MS, Proteomics, Paediatric respiratory disease, Sample preparation, Child, Bronchoscopy, Biomarker discovery

## Abstract

**Background:**

Bronchoalveolar lavage fluid (BALF) is a valuable diagnostic and research tool in paediatric respiratory medicine. Mass spectrometry-based proteomic analysis of BALF can contribute to uncover disease mechanisms and biomarkers, but standardized protocols for paediatric BALF are lacking. This study aimed to establish a robust, reproducible workflow for liquid chromatography-tandem mass spectrometry (LC-MS/MS) of paediatric BALF and to evaluate its applicability in samples from patients with different clinical conditions.

**Methods:**

BALF was collected from five children (ages 1–6 years) undergoing bronchoscopy for various indications. As a reference, we used an adult-derived workflow for BALF analysis, which combines ultracentrifugation (UC) and protein depletion. To address the lower protein yield in paediatric BALF and simplify the procedure, individual steps were systematically omitted, resulting in four workflows: UC plus depletion, UC only, depletion only, and a simplified workflow omitting both. All samples underwent 3 kDa ultrafiltration followed by protein digestion using the S-Trap methodology. Proteins were identified and quantified by LC-MS/MS on an Orbitrap Fusion Lumos Tribrid mass spectrometer. Reproducibility was assessed using technical replicates and BALF from all five patients was analysed to demonstrate applicability. Quantified proteins were further explored by Gene Ontology annotation and pathway mapping.

**Results:**

Altogether, the four workflows quantified 635 proteins from digests standardized to 10 µg protein, readily obtained from as little as 1 ml BALF. Among individual workflows, the simplified workflow yielded the highest number of proteins, with 632 quantified in at least one patient. A core set of 425 proteins (75%) was consistently detected across all patients, regardless of diagnosis. The distribution of coefficient of variation across technical replicates was comparable between workflows. Notably, the simplified workflow reduced hands-on time by approximately five hours compared to the others. Many identified proteins were associated with salivary secretion, complement and coagulation cascades, and Intestinal immune network for IgA production.

**Conclusions:**

This study establishes an efficient and reproducible workflow for proteomic analysis of paediatric BALF requiring smaller sample volumes than typically available from adults. The simplified workflow achieved robust proteome coverage while minimizing sample loss, providing a practical basis for large-scale proteomic studies in paediatric respiratory diseases.

**Supplementary Information:**

The online version contains supplementary material available at 10.1186/s40348-025-00205-0.

## Introduction

Bronchoalveolar lavage (BAL) is a pivotal diagnostic and research tool in respiratory medicine, offering a minimally invasive means to sample the cellular and soluble components of the lower respiratory tract [1, 2]. While serum diffusion contributes to the BAL fluid (BALF) proteome, several proteins are found in higher abundance in BALF, indicating local secretion by alveolar and airway epithelium [3]. This makes BALF a particularly relevant fluid for studying lung diseases, as it directly reflects the injured compartment.

In paediatric populations, BAL is valuable for diagnosing and understanding a range of pulmonary conditions, from infections to inflammatory and interstitial lung diseases [4]. As a reflection of the lung’s microenvironment, BALF contains a rich proteome that can provide insights into disease mechanisms, potential biomarkers, and therapeutic targets [5].

Beyond pathogen detection, BALF enables cellular differentiation, inflammatory profiling, and specialized marker detection. It aids in diagnosing interstitial lung disease [6, 7], quantifying lipid-laden alveolar macrophages [8], identifying respiratory complications in paediatric oncology [9], and probing host-microbe interactions and inflammation in cystic fibrosis via proteomics [10–12]. Reference values for healthy children provide crucial diagnostic baselines [13].

Paediatric BALF composition differs from that of adults, displaying variations such as higher phospholipid concentrations in younger children, consistent protein levels across ages [14], greater epithelial lining fluid recovery [14, 15], higher cell counts and neutrophil percentages in very young children [16], comparable BALF immunoglobulins despite lower serum levels [17], and age-dependent surfactant protein variation [18].

Proteomics, particularly via advanced mass spectrometry techniques such as liquid chromatography-tandem mass spectrometry (LC-MS/MS) offers high sensitivity, specificity, and thorough protein identification, even in complex or low-volume samples [19]. Nevertheless, such paediatric LC-MS/MS applications remain rare; most standardized workflows are still derived from adult BALF protocols [20, 21].

In proteomic experiments, sample preparation is critical, as it underpins the quality and reproducibility of the data by influencing protein recovery, detection sensitivity, susceptibility to artificial modifications (e.g., oxidation or chemical artifacts), sample loss during handling, and analytical bias [22–24]. In paediatric BALF, additional challenges may arise from the inherently small sample volumes and variable protein concentrations [21]. A robust preparation strategy is therefore essential to balance depth of analysis with sample preservation and fidelity.

For adult BALF, protocols are well established [20]; however, systematic adaptations for paediatric samples that address the above challenges remain scarce in the literature. As ethical constraints limit the availability of paediatric BALF [25], emphasizing the imperative for protocols that are efficient, resource-conscious, and reliable in small volumes.

In this study, we apply a streamlined adaptation of an adult BALF LC-MS/MS workflow to paediatric samples. We detail critical steps—from sample collection to protein extraction, cleanup, and LC-MS/MS analysis. We also present proteomic profiles from five paediatric patients with and without pulmonary disease, aiming to establish a reproducible, minimally invasive framework that facilitates high-quality proteomic analysis in paediatric BALF and supports future research in paediatric lung disease.

## Materials and methods

### Subjects

To assess the variability of BALF in children, samples were collected from five paediatric patients over a three-month period. The patients underwent bronchoscopy with BAL for various clinical indications. Their ages ranged from 1 year and 3 months to 6 years and 8 months. Further patient details are provided in Table 1.

**Table 1:** Patient characteristics

ID	Sex	Age (Years)	Indication for bronchoscopy	Main diagnosis	BAL Location	BAL Recovery
A	f	$\:5+\frac{3}{12}$	Follow-up evaluation after foreign body removal (almond)	None	LLL	60%
B	m	$\:6+\frac{6}{12}$	Extended pulmonary diagnostics for immunodeficiency	APDS2	ML	70%
C	f	$\:5+\frac{2}{12}$	Follow-up evaluation after foreign body removal (hazel nut)	None	RLL	70%
D	m	$\:6+\frac{8}{12}$	Recurrent pneumonia with the need for oxygen supplementation	Severe allergic bronchial asthma, chronic rhinopathy, recurrent otitis	ML	50%
E	m	$\:1+\frac{3}{12}$	Combined upper gastrointestinal endoscopy for investigation of wet/gurgly breathing	Unspecified developmental delay	ML	60%

*ADPS2* Activated PI3K delta syndrome type 2, *BAL* Bronchoalveolar Lavage, *LLL* Left Lower Lobe, *ML* Middle Lobe, *RLL* Right Lower Lobe

### BALF collection

Bronchoscopies were performed as in-patient procedures in the endoscopy suite of our intensive care unit; length of the stay was usually one night.

Bronchoscopy and BAL were performed according to international standards [26]. BAL was performed at the segmental site most relevant to the clinical indication. The bronchoscope was placed in a wedge position and triple lung irrigation with warmed saline solution was performed (1 ml per kg body weight). Recovery volumes ranged between 50% and 80%, depending on patient and clinical condition, resulting in yields of approximately 6–50 ml BALF.

BALF was sent to our laboratories for microbiological cultures, respiratory virus multiplex PCR, fungal analysis, molecular genetics for atypical pneumonia pathogens, and FACS analysis of cell components. For routine diagnostic procedures, a minimum of 8 ml BALF is typically required. To ensure sufficient volume for proteomic analysis, the remains of all three BALF aliquots were pooled and utilized for the present study. Separate analysis of lavage fractions was not performed. In patients A-D, 10 ml BALF could be acquired for the study, in patient E 5 ml.

### Preliminary handling of BALF

BALF samples were supplemented with 1 tablet of protease inhibitor per 10 mL (*cOmplete* Mini EDTA-free protease inhibitor tablets, Roche, Basel Swiss), and placed immediately on ice. Samples were vortexed and centrifuged at 4 °C for 15 min at 470 g, resulting in a visible solid pellet, which we assume to represent the cellular fraction of the BALF (predominantly alveolar macrophages, lymphocytes, epithelial and inflammatory cells) as described previously [13]. The pellet is intended for further analyses (e.g., microbiome analysis), and was frozen at −80 °C.

For biosafety reasons, the supernatant was heat-treated at 99 °C for ten min prior to further processing.

After sterilization, the BALF supernatant was frozen and kept at −80 °C.

### Sample preparation workflow

**Fig. 1:**
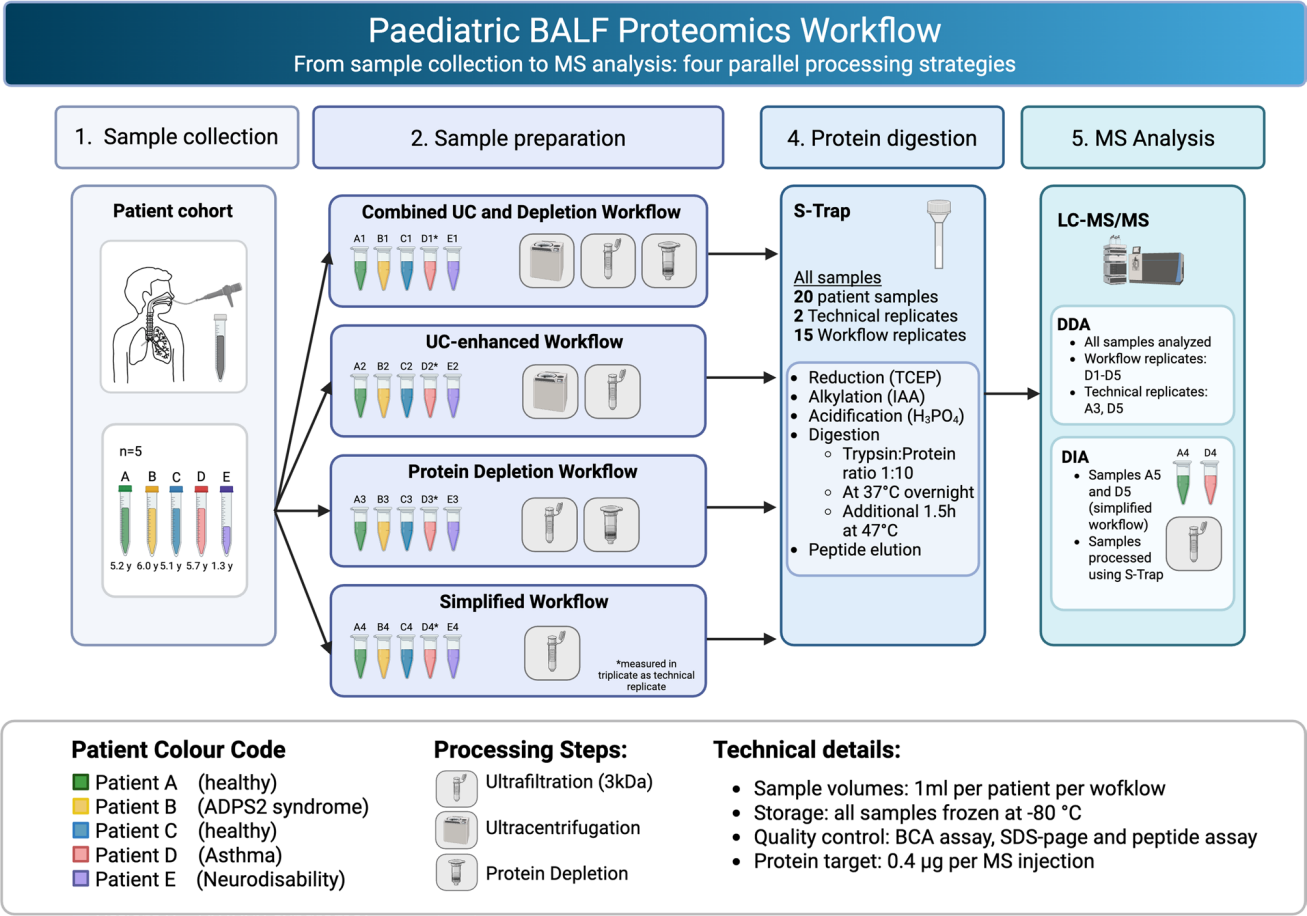
Paediatric BALF proteomics workflow design and sample processing strategy. Comprehensive workflow showing sample collection from five paediatric patients (A-E) through four panel sample preparation approaches: Combined UC and Depletion, UC-enhanced, Protein Depletion, and Simplified workflows. Color coding indicates individual patients with clinical conditions as shown in legend. UC, Ultracentrifugation; BALF, Bronchoalveolar lavage fluid; DIA, data-independent acquisition

Based on an optimized protocol for adult BALF samples [20], we adapted the workflow for paediatric BALF and further simplified it. We systematically compared four approaches: the original workflow (combining ultracentrifugation and high-abundance protein depletion), ultracentrifugation alone, depletion alone, and a simplified protocol omitting both major steps. For each workflow, 1 mL of BALF per patient was processed. Figure 1 illustrates the allocation of patient aliquots to the respective workflows.

#### Ultracentrifugation

BALF samples were centrifuged at 100.000 g for 1h at 4 °C, and the supernatant was used for further sample preparation.

#### Ultrafiltration

BALF or supernatants from ultracentrifugation (UC) were further concentrated using preconditioned (500 µl 5% v/v MeOH in LC-MS grade water) Amicon Ultra 0.5 centrifugal filters (3 kDa MWCO, Merck/Millipore, Darmstadt, Germany). After centrifugation at 14.000 g for 20 min the protein was recovered from the filter reservoir, transferred to protein low-bind tubes, and stored at −80 °C until further use.

#### Depletion

In this step, depletion of 14 high-abundant plasma proteins was performed using High Select mini depletion spin columns (Thermo Scientific) according to the manufacturer’s instructions. In the protein depletion workflow, concentrated BALF was used directly and in the combined UC and depletion workflow samples were used after both ultracentrifugation and concentration by ultrafiltration.

#### Technical replicates

To assess the technical reproducibility of the established procedure, patient D was selected for additional analyses. For each of the four workflows, three independent technical replicates were generated from this patient’s sample, resulting in a total of twelve replicate measurements. These replicates enabled the evaluation of intra-workflow consistency and provided an estimate of the methodological robustness.

#### Protein quantification and visualization

After each processing step, BALF protein concentrations were assessed using the BCA Protein Assay (Pierce BCA Protein Assay Kit, Thermo Scientific). As a visual quality control, proteins were separated by SDS-PAGE and stained with Coomassie Brilliant Blue.

#### Protein purification and digestion

For S-Trap sample processing (ProtiFi, Fairport, United States), samples were diluted in 46 µL sodium dodecyl sulfate (SDS; 5% w/v in water) buffer. Based on BCA assay results, 10 µg of protein (or the whole sample, if less than 10 µg were present) were used for further processing. Where necessary, samples were adjusted to a total volume of 46 µL with SDS buffer (5% v/v in water). Proteins were reduced with 2 µL 500 mM tris(2-carboxyethyl)phosphine hydrochloride in water (TCEP; final concentration 21 mM) for 10 min at 55 °C, followed by alkylation with 6.7 µL of 300 mM Iodoacetamide in water (IAA, final concentration 40 mM) for 20 min in the dark. Protein denaturation was completed by acidifying the sample to pH < 1 using 5.6 µl phosphoric acid (55% v/v) with pH verification by indicator paper. Subsequently, 165 µL of washing buffer (100 mM TEAB and 90% v/v methanol in water) were added, and the solution was transferred to the S-Trap column.

Denatured, non-digested proteins were bound to the S-Trap via centrifugation at 400 g for 30 s. Captured proteins were washed fully free of all contaminants by four consecutive spins at 400 g for one min with 400 µL washing buffer (see above). For tryptic digestion, 0.5 µg trypsin (SERVA, Heidelberg, Germany) suspended in 20 µl (50 mM TEAB v/v in water) was added to each S-Trap column for overnight incubation at 37 °C, followed by an additional 0.5 µg trypsin suspended in 20 µl (50 mM TEAB v/v in water) and a further 1.5 h incubation at 47 °C. According to the S-Trap protocol, a trypsin-to-protein ratio (w/w) of 1:10 was used, however, in samples with protein concentrations below the detection limit of the BCA assay, the actual ratio may have been higher. Digested proteins were sequentially eluted from the S-Trap column using a series of buffers to maximize peptide recovery. Initially, 40 µL of 50 mM TEAB were added and columns were centrifuged at 4,000 g for 1 min to wash out residual digestion buffer. This was followed by the addition of 40 µL of 0.2% trifluoroacetic acid (TFA), and the column was centrifuged again at 4,000 g for 1 min. To enhance the recovery of hydrophobic peptides, 40 µL of 50% acetonitrile with 0.2% (v/v) TFA were used, followed by centrifugation under the same conditions. Finally, 40 µL of 1% (v/v) TFA were added to elute any remaining peptides, with a final centrifugation at 4,000 g for 1 min. All eluates were pooled and dried down using a SpeedVac.

#### Peptide assay

Peptides were quantified using the Pierce Quantitative Colorimetric Peptide Assay (Thermo Fisher Scientific) at 480 nm.

#### LC-MS/MS

A total of 300 ng tryptic peptides were resuspended in 0.2% (v/v) TFA and 2% (v/v) Acetonitrile.

For the LC-MS acquisition an Orbitrap Fusion Lumos Tribrid Mass Spectrometer coupled to an Ultimate 3000 Rapid Separation liquid chromatography system (Thermo Fisher Scientific) equipped with an Acclaim PepMap 100 C18 column (75 μm inner diameter, 25 cm length, 2 μm particle size from Thermo Fisher Scientific) as separation column and an Acclaim PepMap 100 C18 column (75 μm inner diameter, 2 cm length, 3 μm particle size from Thermo Fisher Scientific) as trap column was used. A LC-gradient of 3 h total length and 2 h active separation was applied as described previously (Brenig et al., 2020). For data-dependent (DDA) analysis survey scans were carried out over a mass range from 375 to 1,500 *m/z* at a resolution of 120,000. The target value for the automatic gain control was set to standard and the maximum fill time was 60 ms. Within a cycle time of 2 s the most intense peptide ions (excluding singly charged ions) were selected for fragmentation. Peptide fragments were analysed in the ion trap using rapid mode. Already fragmented ions were excluded for fragmentation for 60 s.

Data analysis was performed with Proteome Discoverer (version 2.4.1.15, Thermo Fisher Scientific), using the standard label-free quantification workflows PWF_Tribrid_Precursor_Quan_and_LFQ_IT HCD_SequestHT_Percolator and CWF_Comprehensive_Enhanced Annotation_LFQ_and_Precursor_Quan, which include the Minora Feature Detector for precursor ion quantification as well as the Percolator node for false-discovery rate derived cut-off determination. RAW files were searched against the human SwissProt database (20,389 entries, UniProtKB, downloaded 12 January 2023) with the MaxQuant 1.6.17.0 contaminant database (Max Planck Institute of Biochemistry, Planegg, Germany), using a precursor mass tolerance of 10 ppm and a fragment mass tolerance of 0.6 Da for fragment spectra. Technical replicates were searched against the Homo sapiens reference proteome (UP000005640, 82,518 entries, UniProtKB, downloaded 8 July 2024). Methionine oxidation, N-terminal acetylation, N-terminal methionine loss and N-terminal methionine loss combined with acetylation were considered as variable modification, carbamidomethylation as static modification. Tryptic cleavage specificity was set to a maximum of two missed cleavage sites. Label-free quantification was performed using standard parameters within the predefined workflow. Post processing, peptide and protein identifications were filtered at a false discovery rate of 1%. Only proteins identified with ≥ 2 unique peptides were considered for quantitative analysis. Contaminants were removed for the analysis. Quality control measures included injection replicates, blank runs to monitor carryover, continuous monitoring of total ion chromatogram and base peak intensity, evaluation of identification rates (PSMs, unique peptides, protein groups), and assessment of retention time reproducibility of commonly detected proteins. Quantification data were normalized using the “Total Peptide Amount” method in Proteome Discoverer to improve comparability.

## Results

### Workflow overview

**Fig. 2:**
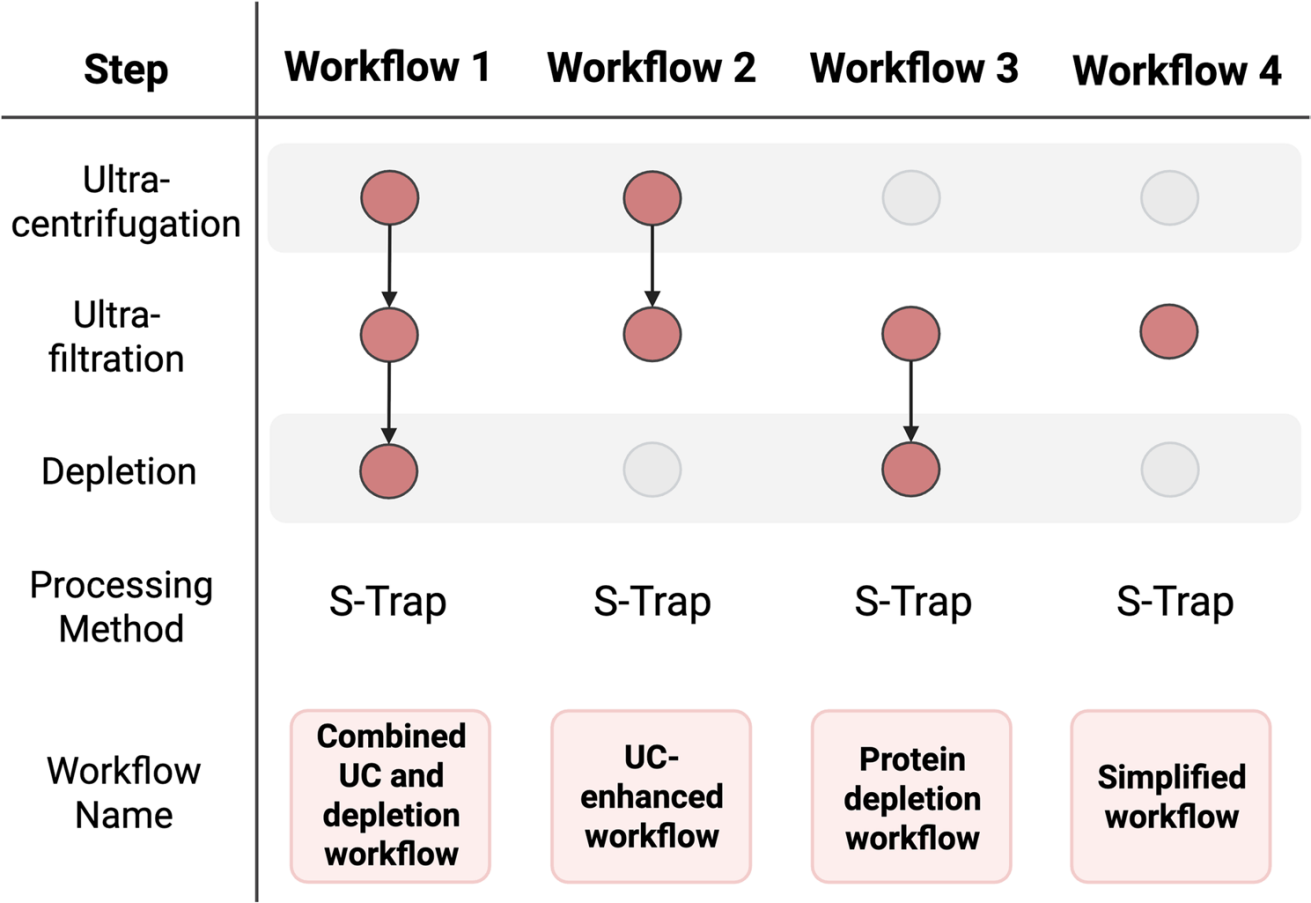
Overview of four distinct sample preparation workflows. Each workflow represents a different combination of ultracentrifugaion, ultrafiltration (3 kDa), and depletion steps, processed using S-trap. The workflows range from the comprehensive combined UC and depletion workflow to the minimal simplified workflow

To establish a processing protocol for paediatric BALF, we adapted an established adult BALF proteomics workflow published by Weise et al. in 2023 [20], which includes initial UC, concentration by ultrafiltration, and depletion of 14 highly abundant proteins. We first applied this combined UC and depletion workflow to our paediatric samples and then systematically compared it with progressively simplified versions designed to minimize protein loss. In these variants, we omitted either the UC step (Protein Depletion workflow), the Top14 depletion step (UC-enhanced workflow), or both steps, which we termed the simplified workflow (Fig. 2).

In our hands, the concentration step using centrifugal filters (3 kDa cut-off) proved essential to enable accurate protein quantification prior to downstream processing. A direct comparison of 3 kDa versus 10 kDa filters showed no substantial difference in protein identification rates.

### Sample preparation efficiency

All workflows - combined UC and depletion, protein depletion, UC-enhanced, and simplified workflow - were feasible to perform with 1 mL of paediatric BALF, which means that even small samples from very young children where BALF volumes are proportionally low can be included in the analysis.

Protein concentrations varied across the different patients, the total protein amount in 1 mL sample ranged between 52.8 µg (patient E) and 342.7 µg (patient D). Average protein recovery after ultrafiltration was 33.7 µg (range from 17.5 to 104.0 µg), with a missing value for some samples of patient E as protein amounts were too little to be detected by the BCA assay. Protein loss analysis revealed substantial differences in recovery efficiency between workflows, with the simplified workflow achieving the highest protein retention (21.6%) followed by the UC-enhanced approach (15.3%), while combined processing steps resulted in recovery rates below 3% (Table 2).

**Table 2:** Protein recovery and loss across processing workflows

		Workflow 1 Combined	Workflow 2 UC-enhanced	Workflow 3 Depletion	Workflow 4 Simplified
Mean initial protein amount ± SD (µg)		155.9 ± 97.8 µg	155.9 ± 111.8 µg	155.9 ± 111.8 µg	155.9 ± 125.0 µg
Mean protein loss per step		-	-	-	-
	UC	63.0%	68.9%	*N/A*	*N/A*
	Ultrafiltration	25.0%	15.8%	85.6%^*b^	78.4%^*a^
	Depletion	11.9%^*c^	*N/A*	11.5%^*b^	*N/A*
Total protein loss (%)		99.9%	84.7%	97.1%	78.4%
Final recovered protein(%)		0.1%	15.3%	2.9%	21.6%

Data presentation: Mean values from patient samples A–E. Loss percentages calculated relative to initial protein amount

Abbreviations: *UC* ultracentrifugation, *BCA* bicinchoninic acid assay, *N/A* not applicable

^a^ Protein concentrations of one sample was below BCA detection level, ^b^ Protein concentration of two samples were below BCA detection level, ^c^ Protein concentrations of three samples were below BCA detection level

The corresponding gel images are provided in the supplementary material (Supplementary Fig. 1).

To evaluate the scalability of our workflows, we estimated the hands-on and total processing time for 30 paediatric BALF samples across all four workflows (*Supplementary Table 1*). The simplified workflow emerged as the fastest and most efficient option, requiring 10–11h of hands-on time and 2 days total processing time, compared to 15–16 h hands-on time and 3 days for the combined UC and depletion workflow. We then analysed each major step of the simplified workflow in detail (*Supplementary Table 2*), revealing that all steps are compatible with process automation, enabling a single technician to complete a 96-sample batch within two working days [27]. This level of throughput would not be feasible in workflows that rely on ultracentrifugation. Although LC-MS/MS run time remains a bottleneck at ~ 3 h per sample, the workflow is compatible with high-throughput strategies such as short-gradient methods on faster scanning mass spectrometers and data-independent acquisition, supporting its applicability in large-scale clinical studies.

### LC-MS/MS Analysis performance

**Fig. 3:**
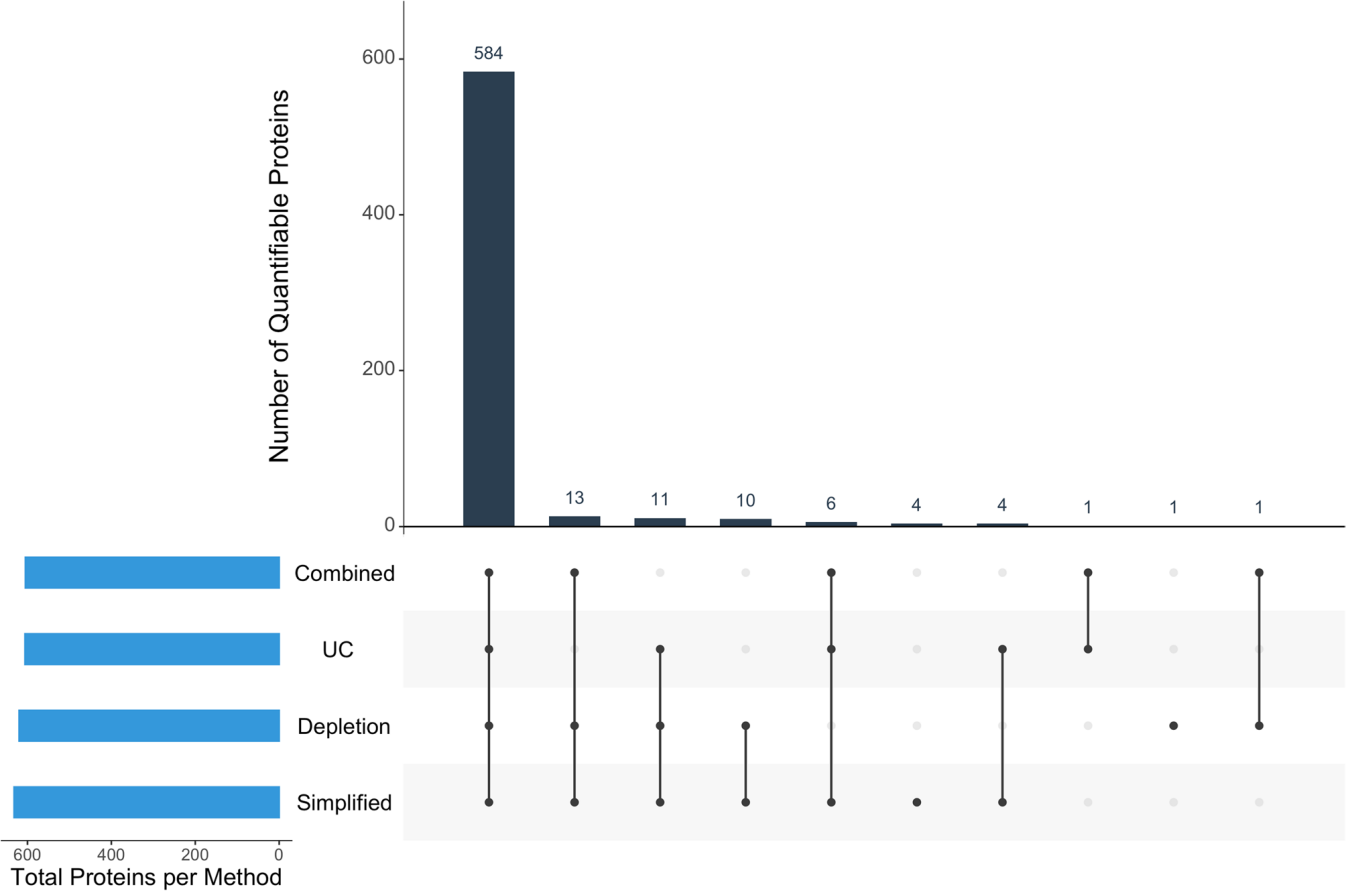
Protein detection overlap between four sample preparation workflows for paediatric BALF proteomics. UpSet plot showing protein detection overlap across the applied four sample preparation methods for paediatric bronchoalvcolar lavage fluid (BALF). Vertical bars indicate the number of proteins detected by specific method combinations, with connected dots below displaying which methods are compared. Horizontal bars show total proteins detected per method. The largest intersection (584 proteins) represents proteins detected by all workflows: Combined (ultracentrifugation+depletion), UC (ultracentrifugation), Depletion and Simplified workflows. The majority of proteins were detected by all four workflows, demonstrating comparable protein detection across preparation methods

A total of 643 proteins were identified across all 20 samples (five patients, four workflows), representing the cumulative number of proteins detected across any of the workflows. Of these, 635 (98.6%) proteins could be quantified in at least one sample. As displayed in Fig. 3, quantitative information was acquired for 584 (91.9%) of these proteins by all four sample preparation methods, demonstrating high consistency across workflows.

The total number of quantifiable proteins varied slightly depending on the workflow, with the simplified workflow detecting the highest number (632 proteins) and the combined UC and depletion workflow identifying the fewest (605). No notable differences were observed in protein molecular weight or protein size across the different workflows. Further details are provided in Table 3.

**Table 3:** Quality of mass spectrometry data

	Combined UC and depletion workflow	UC-enhanced workflow	Protein depletion workflow	Simplified workflow
Number of quantified proteins*	605	606	620	632
Protein size [mean#AA ± SD]	484.76 ± 869.13	485.09 ± 868.65	488.1 ± 861.15	486.49 ± 855.26
Protein weight [MW in kDa]	53.05	53.08	53.45	53.29

Comparison of protein quantification performance across four different mass spectrometry workflows (Combined UC and depletion, UC-enhanced, Protein depletion and simplified worfklows)

Abbreviations: *UC* ultracentrifugation, *AA* amino acids, *MW* molecular weight, *kDa* kilodaltons, *SD* standard deviation

*quantified in at least one of the five samples (Patient A-E)

#### Technical replicates

**Fig. 4:**
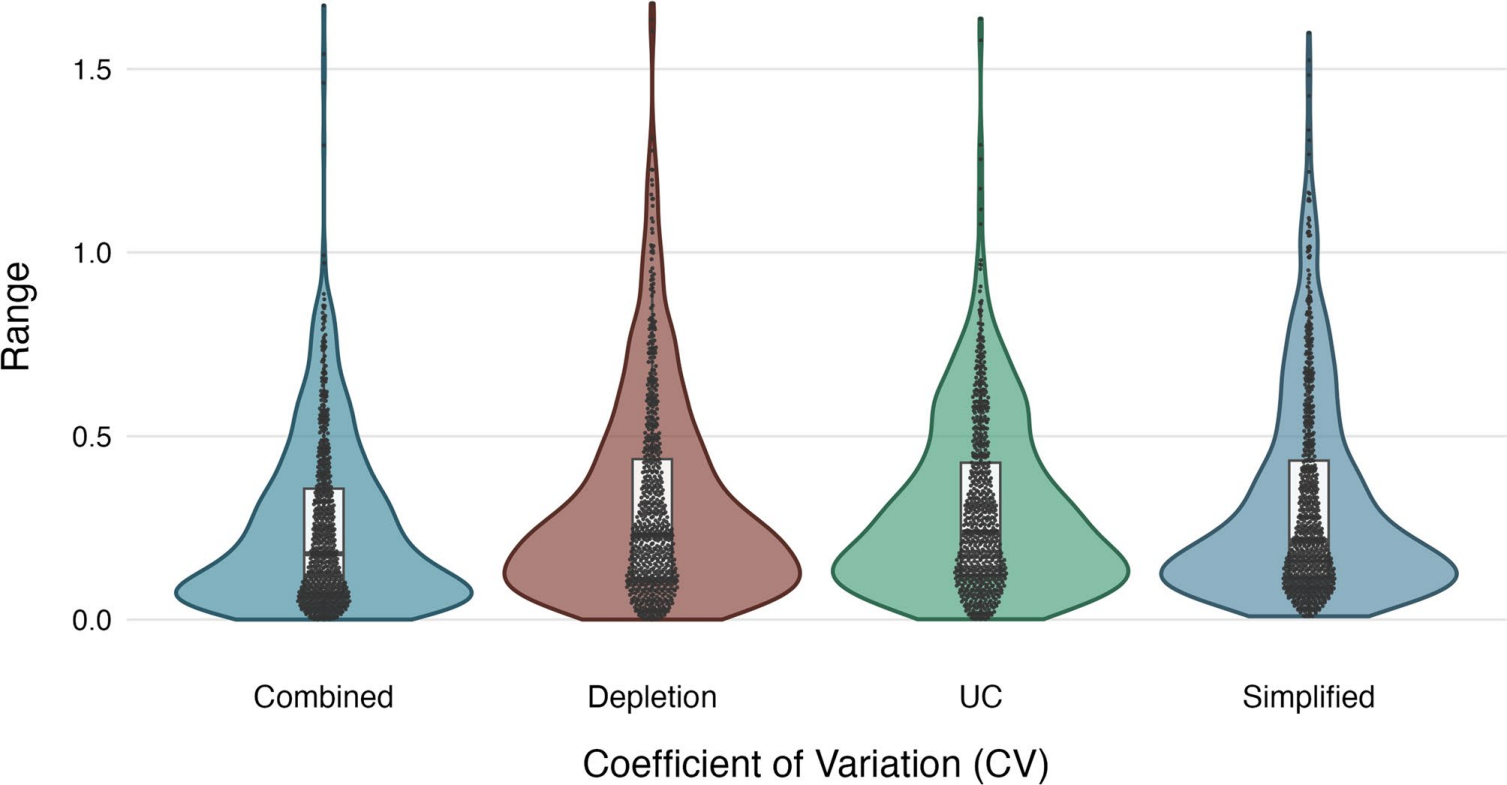
Distribution of coefficient of variation (CV) across sample preparation workflows. Violin plots show the spread of CV values for combined, depletion, ultracentrifugation (UC), and simplified workflows. Box plots within violins indicate median and interquartile ranges, while individual points represent individual measurements

One patient sample was used for individually processing of three replicates per workflow. Resulting samples were analysed using LC-MS/MS, and the consistency between replicates was assessed using Coefficient of variation (CVs) (Fig. 4).

The distribution of CVs across technical replicates was comparable between the four workflows, with median values of 0.32 (Combined), 0.36 (Depletion), 0.42 (UC), and 0.43 (Simplified). Overall, median CVs fell within a similar range, indicating consistent reproducibility across approaches.

#### Protein profiles of individual patients

**Fig. 5:**
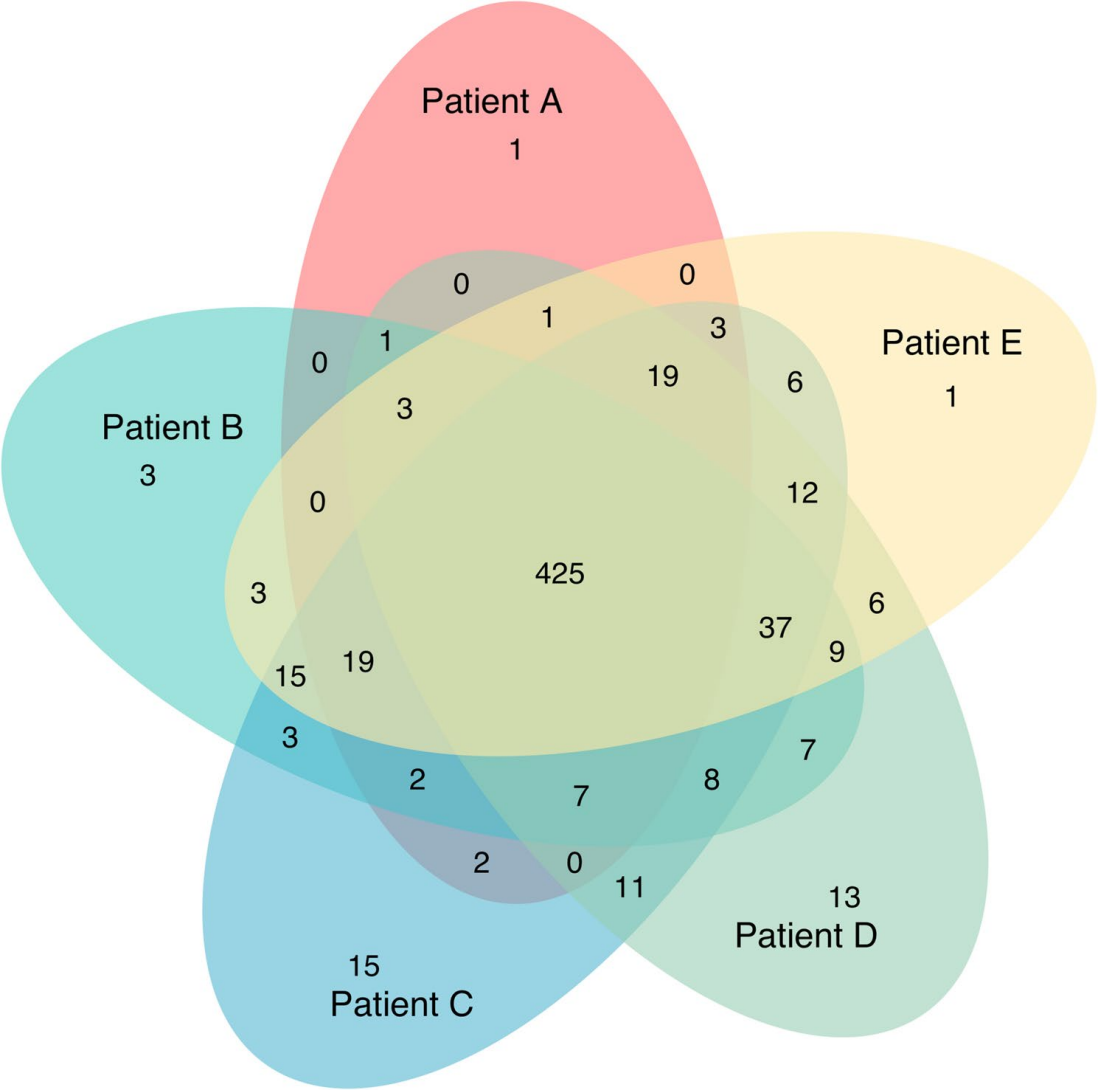
Venn diagram showing protein distribution across five patients with a core of 688 shared proteins. Samples were processed by the simplified workflow. The analysis revealed a total of 592 proteins quantifiable in at least one patient, with 425 (71.8%) proteins quantifiable in all five patients. This substantial core proteome suggests a highly conserved protein composition in BALF, independent of the underlying pathology. Patient-specific proteins ranged from 1 to 15 unique proteins per patient. BALF, Bronchoalveolar lavage fluid

A workflow for paediatric BALF processing should be broadly applicable; therefore, we compared protein profiles from five different patients. The Venn diagram (Fig. 5) illustrates the overlap and distinctiveness of proteins among patients. Overall, the diagram demonstrates a substantial overlap of proteins across all patients indicating a high level of homogeneity of present proteins within the samples despite different underlying diseases.

#### Functional annotation of BALF proteins from individual patients

**Fig. 6:**
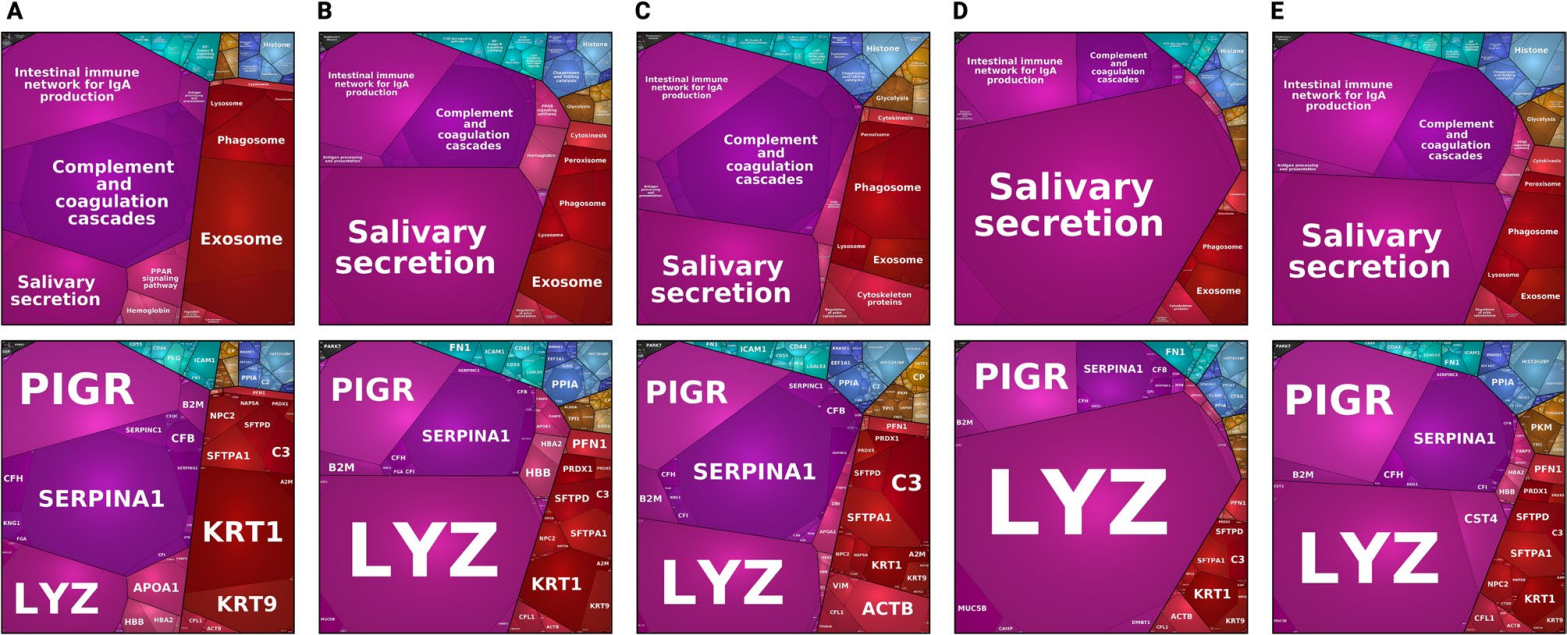
Proteomic profiles of bronchoalveolar lavage fluid (BALF) samples from all patients visualised using Proteomaps. Proteomaps provide a functional overview of the proteomes from each of the five patients (A-E) based on the simplified workflow. It categorises proteins into biological pathways and processes based on their abundance and annotation. Enriched functional pathways (top row) and individual proteins driving those pathways (bottom row) are shown by polygons. Areas of polygons illustrate protein normalized abundance in each region. Functions and proteins linked are organized in common regions and coded using similar colours. Prominent abbreviations: PIGR – Polymeric immunoglobulin receptor, SERPINA1 – Alpha-1-antitrypsin, LYZ – Lysozyme C, KRT – Keratin, C3 – Complement component 3, ACTB – Beta-actin, CFB – Complement

Using protein intensities obtained from the simplified workflow, we generated Proteomaps of BALF samples from all five patients *(*Fig. 6*)*. This approach provides a functional overview of the quantified proteins and pathway-level insights [28, 29]. Prominent pathways included *Salivary secretion*, *Complement and coagulation cascades*, and *Intestinal immune network for IgA production*.

The enrichment of *Salivary Secretion* was largely driven by the high abundance of lysozyme C (LYZ), which was consistently among the most dominant proteins detected. LYZ is a potent bacteriolytic enzyme abundantly present in macrophages—including alveolar macrophages, where it contributes significantly to innate pulmonary defense—by hydrolyzing bacterial cell walls and promoting extracellular bacterial killing [30]. In addition, polymeric immunoglobulin receptor (PIGR) and alpha-1-antitrypsin (SERPINA1) were recurrently present at high levels. Complement proteins (e.g., C3, CFB) and epithelial structural proteins (e.g., KRT1, KRT9) were also detected, though their relative abundance varied between patients.

The ten most abundant proteins per patient, including recurrent detection of lysozyme C, polymeric immunoglobulin receptor, and α1-antitrypsin, are summarized in *Supplementary Table 3*.

In summary, the simplified workflow yielded reproducible proteomic profiles from paediatric BALF, with abundant proteins and pathway enrichments consistent across patients and comparable to previous findings in human BALF.

## Discussion

This study demonstrates that comprehensive proteomic analysis of paediatric BALF is feasible from minimal sample volumes using LC-MS/MS. By systematically comparing four workflows adapted from adult protocols, we identified a simplified approach—omitting both ultracentrifugation and high-abundance protein depletion—that provided the best balance of protein recovery, proteome depth, and reproducibility. Importantly, this workflow enabled the identification of more than 600 proteins from just 1 mL of BALF, establishing a practical and efficient protocol for future clinical and translational studies.

A central finding was that the simplified workflow achieved the highest proteome coverage and protein retention, while additional processing steps such as ultracentrifugation or depletion resulted in considerable protein loss without clear benefit. In our samples, the average protein recovery after ultrafiltration was approximately 30 µg, whereas adult BALF samples processed with a similar step [20] yielded around 260 µg, highlighting the markedly lower protein availability in paediatric material. Weise et al. employed depletion and ultracentrifugation to reduce abundant plasma proteins or enrich vesicular fractions, but our data show that in paediatric BALF, the drawbacks of protein loss outweigh potential gains in sensitivity. From a methodological standpoint, omitting these steps minimizes handling, reduces variability, and preserves protein yield—an essential consideration when working with the small and often variable volumes obtainable in children.

Across the five patients, 688 proteins (approximately 75% of all detected proteins) were identified in common. This substantial overlap indicates that a large proportion of the BALF proteome is shared among children despite differing clinical conditions, highlighting the need for quantitative analyses to uncover disease-specific differences or biomarkers. These findings also demonstrate that reproducible protein profiles can be obtained from paediatric BALF, with a consistent subset of proteins detectable across patients. BALF collected after foreign body removal may furthermore serve as a practical reference material in the absence of true healthy controls, although this will require further validation. Previous studies in single disease entities [31, 32] support the feasibility of detecting disease-specific proteomic signatures, but more comprehensive multi-disease analyses will be required.

When comparing absolute protein yields with recent adult BALF proteomics studies, it is clear that methodological and sample differences strongly influence reported protein numbers. For example, Weise et al. identified ~ 988 proteins in unfractionated BALF and up to ~ 1,800 with TMT multiplexing and fractionation [20], while Tharp et al. reported ~ 1,045 proteins using SWATH/DIA on larger adult BALF volumes [33]. Mitsuyama et al. achieved the deepest coverage, with ~ 2,000 proteins detected in ARDS patient BALF using DIA and higher input volumes [34]. In contrast, our protocol was deliberately optimized for minimal paediatric input (1mL BALF) and single-shot DDA, yet reproducibly yielded >600 proteins. This represents fewer proteins than high-depth adult studies, but within the expected range given input and acquisition constraints, and importantly, we demonstrate that robust and reproducible proteomes can be obtained from very limited paediatric material.

Beyond analytical performance, we evaluated the scalability of the workflows in terms of time and personnel requirements. The simplified workflow proved not only the most efficient in terms of proteome recovery but also the least demanding in terms of processing time, requiring approximately 10–11 h of hands-on time and two working days for a batch of 30 samples. This finding is particularly relevant for future large-scale studies, where feasibility and throughput are critical. Taken together, these characteristics support the feasibility of larger cohorts, which are essential for establishing reference data sets and exploring disease-specific alterations.

The research potential of this protocol extends beyond diagnostic applications: Integration with other molecular techniques, such as metabolomics or transcriptomics, could provide more comprehensive insights into paediatric lung pathophysiology, an approach that has shown promise in adult studies [31].

Several limitations must be acknowledged. First, the small number of patients limits the generalizability of our findings and precludes disease-specific conclusions. Second, aliquots from sequential lavage fractions were pooled after BAL to maximize available volume, which precluded analysis of potential differences between proximal and distal fractions or distinct anatomic regions. Future studies should explore whether such stratification provides additional diagnostic or biological information. Third, all samples underwent mandatory heat inactivation for biosafety, which may have altered protein integrity. Since this treatment was applied uniformly, relative comparisons remain valid, but comparative experiments without heat treatment would be informative. Finally, variability in BALF recovery and dilution remains an inherent challenge and is not resolved by the present protocol [35]. Careful documentation and harmonization of collection parameters will be critical for multicentre applications.

## Conclusions

In summary, we provide the first systematic evaluation of BALF sample preparation workflows in children and establish a simplified, reproducible protocol optimized for low input volumes. This approach enables high-quality proteomic analysis from routine clinical samples and is scalable to larger cohorts. With further validation, standardized BALF proteomics has the potential to advance our understanding of paediatric respiratory diseases and contribute to biomarker discovery.

## Supplementary Information

Supplementary Material 1. Supplementary Table 1. Time requirements for all four paediatric BALF proteomics workflows Overview of active technician time (for 30 samples) and total processing time from sample thawing to ready-for-MS samples across four different workflows: combined ultracentrifugation (UC) and depletion, UC alone, depletion alone, and simplified workflow. LC-MS/MS run time is not included. Supplementary Table 2. Time requirements and scalability assessment for paediatric BALF proteomics simplified workflowDetailed breakdown of hands-on time, total time, batch size, and automation potential for each step of the simplified workflow. Estimates are based on processing 96 samples, excluding LC-MS/MS run time. Supplementary Table 3. Top 10 most abundant proteins across five patientsProteins are ranked by abundance for each patient (A–E). UniProt accession numbers are provided. Shared and patient-specific proteins are highlighted.

Supplementary Material 2. Supplementary Figure 1. SDS-PAGE analysis of protein samples.Proteins were separated on 4–12% gradient polyacrylamide gels and visualized using Coomassie Brilliant Blue staining. The molecular weight marker is shown in the left lane of each gel, with molecular weights (in kDa) indicated. BALF samples from five patients (A–E) were processed according to the four tested workflows: (i) combined UC and depletion workflow, (ii) UC-enhanced workflow, (iii) protein depletion workflow, and (iv) simplified workflow. Each lane corresponds to 1 mL BALF processed according to the respective protocol. Gels were run after each step of the protocol to visualize protein recovery and loss across workflows. Additional samples from patient A and patient D were processed in parallel to check consistency, but these were not included in the final proteomic analysis. The lower two gels illustrate the protein depletion workflow, showing protein content after passage through 3 kDa molecular weight filters before depletion (left) and after depletion of 14 abundant proteins (right). The gels highlight the marked reduction in protein bands following combined processing (UC + depletion), in contrast to the broader protein retention observed with the simplified workflow.

## Data Availability

The mass spectrometry proteomics data have been deposited to the ProteomeXchange Consortium via the PRIDE partner repository [doi: 10.1093/nar/gkae1011] with the dataset identifier PXD067840. The mass spectrometry proteomics data have been deposited to the ProteomeXchange Consortium via the PRIDE partner repository [36] with the dataset identifier PXD067840.

## Abbreviations

BALF: Bronchoalveolar Lavage Fluid
BAL: Bronchoalveolar Lavage
LC-MS/MS: Liquid Chromatography-Tandem Mass Spectrometry
UC: Ultracentrifugation
S-Trap: Suspension Trapping
SP3: Single-pot, Solid-phase-enhanced sample Preparation
GO: Gene Ontology
CV: Coefficient of Variation
SD: Standard Deviation
PSM: Peptide Spectrum Matches
DDA: Data-Dependent Analysis
DIA: Data-Independent Analysis
kDa: Kilodalton
MWCO: Molecular Weight Cut-Off
PBS: Phosphate-Buffered Saline
SDS-PAGE: Sodium Dodecyl Sulfate Polyacrylamide Gel Electrophoresis
APDS2: Activated PI3K Delta Syndrome type 2
FDR: False Discovery Rate
TEAB: Triethylammonium Bicarbonate
TFA: Trifluoroacetic Acid
ACN: Acetonitrile
DTT: Dithiothreitol
IAA: Iodoacetamide
MW: Molecular Weight
