# Supplementary material for: Standardized sample preparation of paediatric bronchoalveolar lavage fluid for mass spectrometry based proteomic analysis
Source: Mol Cell Pediatr. 2025 Nov 20;12:21. doi: 10.1186/s40348-025-00205-0 (PMC12634934; doi:10.1186/s40348-025-00205-0)
Supplement: Supplementary file 1 — Supplementary Material 1. Supplementary Table 1. Time requirements for all four paediatric BALF proteomics workflows Overview of active technician time (for 30 samples) and total processing time from sample thawing to ready-for-MS samples across four different workflows: combined ultracentrifugation (UC) and depletion, UC alone, depletion alone, and simplified workflow. LC-MS/MS run time is not included. Supplementary Table 2. Time requirements and scalability assessment for paediatric BALF proteomics simplified workflowDetailed breakdown of hands-on time, total time, batch size, and automation potential for each step of the simplified workflow. Estimates are based on processing 96 samples, excluding LC-MS/MS run time. Supplementary Table 3. Top 10 most abundant proteins across five patientsProteins are ranked by abundance for each patient (A–E). UniProt accession numbers are provided. Shared and patient-specific proteins are highlighted. [file 40348_2025_205_MOESM1_ESM.docx]

**Supplementary Table 1. Time requirements for all four paediatric BALF proteomics workflows**

Overview of active technician time (for 96 samples) and total processing time from sample thawing to ready-for-MS samples across four different workflows: combined ultracentrifugation (UC) and depletion, UC alone, depletion alone, and simplified workflow. LC-MS/MS run time is not included.

| **Supplementary table S1.** Time requirements for all four paediatric BALF proteomics workflows | | | | |
| --- | --- | --- | --- | --- |
| **Workflow** |  | **Total Hands-on Time^a^** |  | **Total Processing Time^b^** |
| Combined UC and depletion workflow |  | 15-16 h |  | 3 working days |
| UC-enhanced workflow |  | 13-14 h |  | 2.5-3 working days |
| Protein depletion workflow |  | 12-13 h |  | 2.5-3 working days |
| Simplified workflow |  | 10-11 h |  | 2 working days |
| ^a^ Active technician time for 96 samples, excluding automated incubations and LC-MS/MS analysis  ^b^ Total processing time from sample thawing to ready-for-MS samples (excludes LC-MS/MS run time)  **Abbreviations**: BALF, bronchoalveolar lavage fluid; LC-MS/MS, liquid chromatography-tandem mass spectrometry; UC, ultracentrifugation | | | | |

**Supplementary Table 2. Time requirements and scalability assessment for paediatric BALF proteomics simplified workflow**

Detailed breakdown of hands-on time, total time, batch size, and automation potential for each step of the simplified workflow. Estimates are based on processing 96 samples, excluding LC-MS/MS run time.

| **Supplementary Table S2.** Time requirements and scalability assessment for paediatric BALF proteomics simplified workflow | | | | | | |
| --- | --- | --- | --- | --- | --- | --- |
| **Workflow step** |  | **Hands-on-Time** |  | **Total Time** |  | **Batch Size** |
| ***Sample Preparation*** | | | | | | |
| Sample thawing |  | 10 min |  | 30 min |  | 96 |
| Sample ultrafiltration (3 spins per sample to prep filters) |  | 1.5–2 h |  | 3–4 h |  | 8–12 (96^a^) |
| Protein recovery spin (Amicon) |  | 15 min |  | 30 min |  | 96^a^ |
| ***Protein Analysis & QA*** | | | | | | |
| Reconstitution + sonication |  | 20 min |  | 20 min |  | 96 |
| BCA protein assay |  | 30 min |  | 1 h (incl. incubation) |  | 96 |
| Gel electrophoresis (QC) |  | 30 min setup |  | 2 h run + overnight fixation |  | 96 (Parallel processing) |
| ***MS Sample Preparation*** | | | | | | |
| SpeedVac drying |  | 10 min setup |  | 3–4 h |  | 96 |
| Protein solubilization |  | 30 min |  | 30 min |  | 96 |
| S-Trap prep, digestion, elution |  | 5-6h |  | Overnight digestion |  | 96 |
| **Total Workflow (69 samples)** |  | **~10–11 h** |  | **2 working days** |  | **30** |
| **Time estimates**: Based on processing 96 paediatric BALF samples in a plate format using the simplified workflow. Hands-on time represents active technician involvement; total time includes incubation and processing delays.  ^a^ Limited by centrifuge rotor capacity (typically 12 × 15 mL tubes), parallel setup possible (filtration plate, positive pressure devide)  ^b^ Minimal hands-on time during automated LC-MS/MS runs  **Abbreviations**: BALF, bronchoalveolar lavage fluid; BCA, bicinchoninic acid assay; LC-MS/MS, liquid chromatography-tandem mass spectrometry; QC, quality control; UC, ultracentrifugation | | | | | | |

**Supplementary Table 3. Top 10 most abundant proteins across five patients**

Proteins are ranked by abundance for each patient (A–E). UniProt accession numbers are provided. Shared and patient-specific proteins are highlighted.

| **Supplementary Table 3. Top 10 most abundant proteins across five patients** | | | | | | | | | | |
| --- | --- | --- | --- | --- | --- | --- | --- | --- | --- | --- |
| **Rank** |  | **Protein Description (UniProt Accession)** | | | | | | | | |
|  |  | Patient A |  | Patient B |  | Patient C |  | Patient D |  | Patient E |
| **1** |  | Serotransferrin (P02787) |  | Serotransferrin (P02787) |  | Serotransferrin (P02787) |  | Lysozyme C (P61626) |  | Lysozyme C (P61626) |
| **2** |  | Α-1-antitrypsin (P01009) |  | Lysozyme C (P61626) |  | IG heavy constant γ 1 (P01857) |  | Serotransferrin (P02787) |  | Serotransferrin (P02787) |
| **3** |  | Polymeric IG receptor (P01833) |  | Polymeric IG receptor (P01833) |  | Lysozyme C (P61626) |  | Lipocalin-1 (P31025) |  | IG heavy constant α 1 (P01876) |
| **4** |  | IG heavy constant γ 1 (P01857) |  | Lactotransferrin (P02788) |  | Α-1-antitrypsin (P01009) |  | Lactotransferrin (P02788) |  | Polymeric IG receptor (P01833) |
| **5** |  | Keratin, type II cytoskeletal (P04264) |  | Α-1-antitrypsin (P01009) |  | Polymeric IG receptor (P01833) |  | Polymeric IG receptor (P01833) |  | Lactotransferrin (P02788) |
| **6** |  | IG heavy constant α 1 (P01876) |  | IG heavy constant γ 1 (P01857) |  | IG heavy constant α 1 (P01876) |  | Keratin, type II cytoskeletal (P04264) |  | Α-1-antitrypsin (P01009) |
| **7** |  | Lysozyme C (P61626) |  | Lipocalin-1 (P31025) |  | A-1-acid glycoprotein (P02763) |  | IG heavy constant α 1 (P01876) |  | IG kappa constant (P01834) |
| **8** |  | Hemopexin (P02763) |  | Keratin, type II cytoskeletal (P04264) |  | Echinoderm microtubule-associated protein-like 6 (Q6ZMW3) |  | Antileukinproteinase (P03973) |  | Antileukinproteinase (P03973) |
| **9** |  | A-1-acid glycoprotein (P02763) |  | IG heavy constant mu (P01871) |  | Lactotransferrin (P02788) |  | IG kappa constant (P01834) |  | Cystatin-S (P01036) |
| **10** |  | Keratin, type I cytoskeletal (P35527) |  | Antileukinproteinase (P03973) |  | Hemopexin (P02763) |  | Α-1-antitrypsin (P01009) |  | IG lambda constant (P0DOY2) |
| Rank order of the ten most abundant proteins identified by LC-MS/MS proteomics in each patient (A-E). Only high-confidence protein identifications were included (FDR-controlled, minimum two peptides per protein). Contaminant proteins were excluded from analysis.  **Abbreviations:** *IG Immunoglobulin* | | | | | | | | | | |
