# Supplementary figures and images for: Standardized sample preparation of paediatric bronchoalveolar lavage fluid for mass spectrometry based proteomic analysis
Source: Mol Cell Pediatr. 2025 Nov 20;12:21. doi: 10.1186/s40348-025-00205-0 (PMC12634934; doi:10.1186/s40348-025-00205-0)

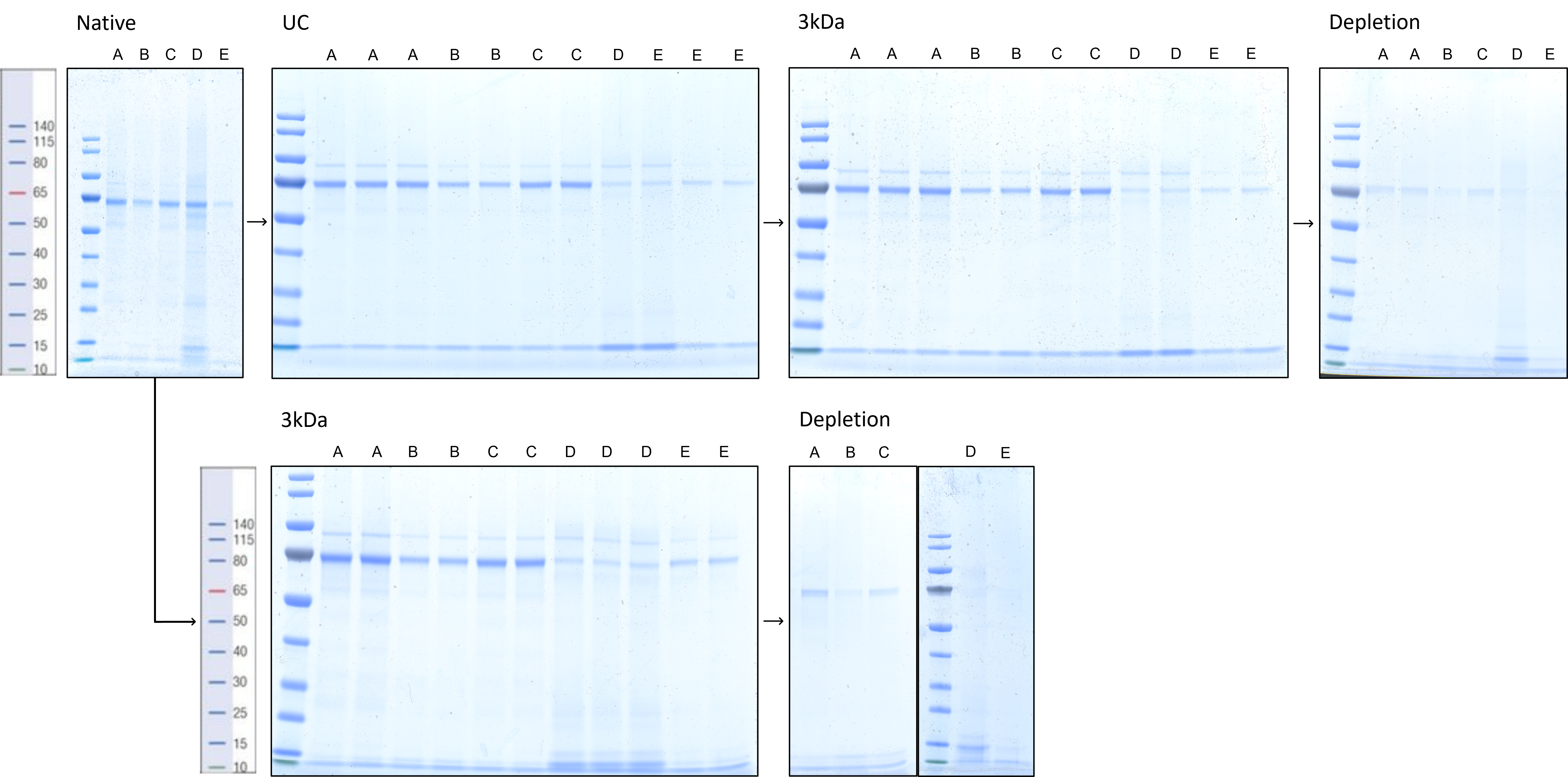

Supplement: Supplementary file 2 — Supplementary Material 2. Supplementary Figure 1. SDS-PAGE analysis of protein samples.Proteins were separated on 4–12% gradient polyacrylamide gels and visualized using Coomassie Brilliant Blue staining. The molecular weight marker is shown in the left lane of each gel, with molecular weights (in kDa) indicated. BALF samples from five patients (A–E) were processed according to the four tested workflows: (i) combined UC and depletion workflow, (ii) UC-enhanced workflow, (iii) protein depletion workflow, and (iv) simplified workflow. Each lane corresponds to 1 mL BALF processed according to the respective protocol. Gels were run after each step of the protocol to visualize protein recovery and loss across workflows. Additional samples from patient A and patient D were processed in parallel to check consistency, but these were not included in the final proteomic analysis. The lower two gels illustrate the protein depletion workflow, showing protein content after passage through 3 kDa molecular weight filters before depletion (left) and after depletion of 14 abundant proteins (right). The gels highlight the marked reduction in protein bands following combined processing (UC + depletion), in contrast to the broader protein retention observed with the simplified workflow. [file 40348_2025_205_MOESM2_ESM.png]
